# Efficacy and safety of basal-bolus insulin at 1:1.5 ratio compared to 1:1 ratio using a weight-based initiation and titration (WIT2) algorithm in hospitalized patients with type 2 Diabetes: a multicenter, randomized, clinical study

**DOI:** 10.1186/s13098-023-01193-9

**Published:** 2023-11-27

**Authors:** Xiaodan Zhang, Dewen Yan, Tao Du, Yunjuan Zhao, Jiangong Zhang, Tong Zhang, Mingrun Lin, Yanli Li, Wangen Li

**Affiliations:** 1 Department of Endocrinology, The Second Affiliated Hospital of Guangzhou Medical University, Guangzhou, China; 2 Department of Endocrinology, The First Affiliated Hospital of Shenzhen University, Shenzhen, China; 3 Department of Endocrinology, The Third Affiliated Hospital of Southern Medical University, Guangzhou, China; 4 Department of Endocrinology, The Fifth Affiliated Hospital of Guangzhou Medical University, Guangzhou, China

**Keywords:** Diabetes, Insulin, Basal-bolus, Initiation, Titration

## Abstract

**Background:**

Most studies initiated basal-bolus insulin in a ratio of 1:1 and titrated based on glucose. This study aimed to investigate the effectiveness and safety of a weight-based and ratio of 1:1.5 basal-bolus insulin using an algorithm for both initiation and titration in hospitalized patients with type 2 diabetes (T2D).

**Methods:**

Hospitalized patients with T2D were randomly assigned to two groups in equal numbers to receive 1:1.5 and 1:1 ratios of basal-bolus insulin using a weight-based algorithm for both initiation and titration. The primary outcome was the time taken to reach the fasting blood glucose (FBG) target and 2-h postprandial blood glucose (2hBG) targets after three meals. The secondary outcome included insulin dosage to achieve glycemic control and the incidence of hypoglycemia during hospitalization.

**Results:**

250 patients were screened between October 2021 and June 2022, 220 were randomly grouped, and 182 completed the trial (89 in the 1:1.5 and 93 in the 1:1 groups). The time taken to reach FBG targets was comparable between the two groups (3.4 ± 1.7 vs. 3.0 ± 1.3 days, p = 0.137) within about 3 days. The 2hBG after three meals was shorter in the 1:1.5 group than in the 1:1group (2.9 ± 1.5 vs. 3.4 ± 1.4 days, p = 0.015 for breakfast, 3.0 ± 1.6 vs. 3.6 ± 1.4 days, p = 0.005 for lunch, and 3.1 ± 2.1 vs. 4.0 ± 1.5 days, p = 0.002 for dinner). No significant difference in insulin dosages was found between the two groups at the end of the study. The incidence of hypoglycemia was similar in both groups.

**Conclusions:**

We demonstrated that fixed dose-ratio basal-bolus insulin at 1:1.5 calculated using a weight-based initiation and titration algorithm was simple, as effective, and safe as ratio at 1:1 in managing T2D in hospitalized patients.

*Trial Registration * ChiCTR 2,100,050,963. Date of registration: September 8, 2021.

## Background

Hospitalized patients with hyperglycemia require a longer hospital stay and face several complications [1, 2]. The glycemic level at fasting (fasting blood glucose, FBG) and 2-h postprandial blood glucose (2hBG) targeting 7.8 and 10.0 mmol/L, respectively, in non-critically ill patients with hyperglycemia, could prevent adverse outcomes [2, 3].

Insulin is superior to other medications for the rapid control of hyperglycemia in hospitalized patients. The normal physiological pattern of insulin secretion by the pancreas consists of basal release and burst of bolus insulin. American Association of Clinical Endocrinologists (AACE) and American Diabetes Association (ADA) recommend basal-bolus insulin instead of slide-scale insulin for glycemic control due to its effectiveness and safety [4]. However, the latest survey revealed that more than 30% of physicians still prefer to use slide-scale insulin in clinical practice because the administration of bolus insulin is complex [5–7]. Many studies simplified the insulin administration regimen, for example, by using basal plus [7], reducing correction insulin administration [8, 9], using premixed insulin [10], or only using basal insulin [11].

In healthy individuals, the ratio of basal and bolus insulin levels is 50:50 [12]. Therefore, when initiating insulin therapy in hospitalized patients with type 2 diabetes (T2D) one-half of the total daily dose (TDD) was given as a basal dose (glargine or determir) once daily at bedtime, and the other half was given as a bolus (aspart, lispo, or glulisine) in equally divided doses before breakfast, lunch, and dinner regardless of TDD calculation based on body mass index (BMI) or random glucose level [2, 13, 14]. At the end of the corresponding clinical trials, Umpierrez found that the ratio of basal-bolus was about 1:1 in their two clinical trials (glargine to glulisine, 22:20 units/day and 43:42 units/day) and Meyer et al. found the ratio as about 1:1 (glargine to glulisine 33:36 units/day) [13–15].

Currently, the initial and final ratio of basal-bolus are kept at 1:1. We also designed a study that had a titrating ratio of 1:1 during the middle period. Surprisingly, when FBG and three 2hBG reached the target levels, the ratio was 1:1.5 (glargine to aspart, 25:36 units/day) [16]. Liu et al. used a continuous subcutaneous infusion of titrated insulin and the study found a ratio of 1:1.5 when the target glucose level was attained [17]. A study with the Latin American non-intensive care unit patients with T2D used basal-bolus and had a ratio of about 1:1.5 (glargine to glulisine, 22:31 units/day) [18].

This multicenter, randomized, controlled clinical study aimed to investigate whether the weight-based, 1:1.5 basal-bolus insulin initiation and titration using an algorithm is superior to 1:1.

### Patients

This multicenter, randomized prospective study was performed in the department of endocrinology of four medical centers (The Second Affiliated Hospital of Guangzhou Medical University, The Third Affiliated Hospital of Southern Medical University, the Fifth Affiliated Hospital of Guangzhou Medical University, and the First Affiliated Hospital of Shenzhen University) between October 2021 and June 2022. Informed consent was obtained from each participant. This study was approved by the ethics Committee of our hospital.

### Inclusion/exclusion criteria

Patients who were aged between 18 and 75 years and diagnosed previously or newly with T2D, with a blood glucose (BG) level of > 10.0 mmol/L on admission were randomly selected for the current study. The patients were excluded if they were in one of the following criteria: (i) patients who received insulin therapy at a daily dosage of > 0.4 U/kg before admission. This is because one of our group initiated insulin 0.4 U/kg, if patient had used a daily dosage of > 0.4 U/kg before admission, their glucose maybe worsen ; (ii) patients who were unable to eat; (iii) patients who received corticosteroid therapy; (iv) patients who had renal insufficiency with the plasma creatinine concentration of ≥ 130 µmol/L or liver insufficiency (aspartate aminotransferase or alanine aminotransferase concentration of ≥ two-fold normal range), this is because insulin was metabolized in liver and kidney; (v) patients who were pregnant; (vi) patients with a previous or current history of malignant tumors.

### Randomization

Randomization codes were generated using a computer program (SPSS V.25.0). Patients were randomly assigned at a ratio of 1:1 to the two treatment groups on the first day of admission at the four medical centers. Neither patients nor investigators were masked to the treatment group.

### Study protocol

Basal insulin bolus consisting of a subcutaneous injection of glargine (Sanofi Aventis Deutschland GmbH, Frankfurt, Germany) at bedtime and aspart (Novo Nordisk, Bagsværd, Denmark) before each of three meals. The blood glucose levels of the patients were checked during the round at 09:00 h and insulin titration dose was determined by physicians based on FBG in the morning and 2hBG after breakfast from the same day and 2hBG after lunch and dinner from the previous day.

All other antidiabetic agents were discontinued on the day of admission. Insulin was initiated at a TDD of 0.5 units/kg in the 1:1.5 group and 0.4 units/kg in the 1:1 group. The 1:1.5 group received 40% (0.2 units/kg) of the TDD as glargine and 60% (0.3 units/kg) as aspart. The 1:1 group received 50% (0.2 units/kg) of the TDD as glargine and 50% as aspart (0.2 units/kg). Glargine was administered as a single daily dose while aspart was divided into three equal parts.

Both glargine and aspart were titrated using a weight-based algorithm. In the 1:1.5 and 1:1 groups, glargine was titrated at 0.1 units/kg/day. In the 1:1.5 group, aspart was titrated at 0.05 units/kg/day, and in the 1:1 group, aspart was titrated at 1/3 of 0.1 units/kg/day before each meal. When one 2hBG level reached the target, the aspart was not titrated further (Table 1). The FBG and 2hBG target levels were set at 7.8 mmol/L and 10.0 mmol/L, respectively, as recommended by the American Endocrine Society, respectively (Table 1) [2]. If hypoglycemia was seen, the corresponding insulin titration was held.

**Table 1:** Insulin initiation and titration using the algorithm

	1:1.5 Glargine	Aspart	1:1 Glargine	Aspart
Initiation	0.2 U/kg	0.1 U/kg	0.2 U/kg	0.2 U/kg×1/3
Titration (day)	+ 0.1 U/kg	+ 0.05 U/kg	+ 0.1 U/kg	+ 0.1 U/kg×1/3
Hypoglycemia	− 0.1 U/kg	− 0.05 U/kg	− 0.1 U/kg	− 0.1 U/kg×1/3

Glargine was titrated based on FBG target level of <7.8 mmol/L; Aspart was titrated based on 2hBG target level of <10.0 mmol/L. U, units

Blood glucose levels at five points were measured, including FBG, 2hBG after three meals, and BG at 03:00 h using a glucose meter (Accu-Chek Advantage; Roche Diagnostics, Basel, Switzerland). Additionally, the glucose levels were measured when patients reported symptoms of hypoglycemia. Hypoglycemia was classified into three categories (level 1:3.0 ≤ BG < 3.9 mmol/L; level 2: BG < 3.0 mmol/L; level 3: a severe event that requires assistance from another person for treatment of hypoglycemia). Hemoglobin A1c (HbA1c) was tested in all patients on day 2 of hospitalization.

All centers ordered dietary profile according to our textbook. First, we calculated ideal weight (kg) which is height (cm) minus 105. Second, we calculated total energy between 25 (overweight or obese) and 30 (normal weight or lean) kcal/kg/day. Third, in our diet, protein was given 1.0 g/kg ideal weight, fat was given 0.8 g/kg ideal weight, and left energy was given as carbohydrate which account for about 50–55% of total energy. Total calories were divided in a ratio of 1:2:2 across the three daily meals.

### Outcome measures

The primary outcome of the study was the time for achieving FBG and 2hBG target levels. The secondary outcome was the incidence of hypoglycemia during hospitalization.

### Statistical analysis

The primary endpoint was considered on the day when 2hBG reached the target level after three meals. Based on our previous study [9], a significant difference in the time was considered when 2hBG reached the target level on any 1 day between the two groups. Assuming significant differences of three points with α = 0.05% and 90% power, the required number of patients for each group was 86. To allow a 20% dropout rate, it needed 215 patients and we recruited 220 patients. The basic characteristics of subjects and outcome variables were compared using an independent *t*-test or χ^2^ test as appropriate. Statistical analyses were performed using the SPSS version 25.0 (SPSS, Chicago, IL). The *p*-value of < 0.05 was considered statistically significant. Data were provided as means ± SD or median (range).

## Results

**Fig. 1:**
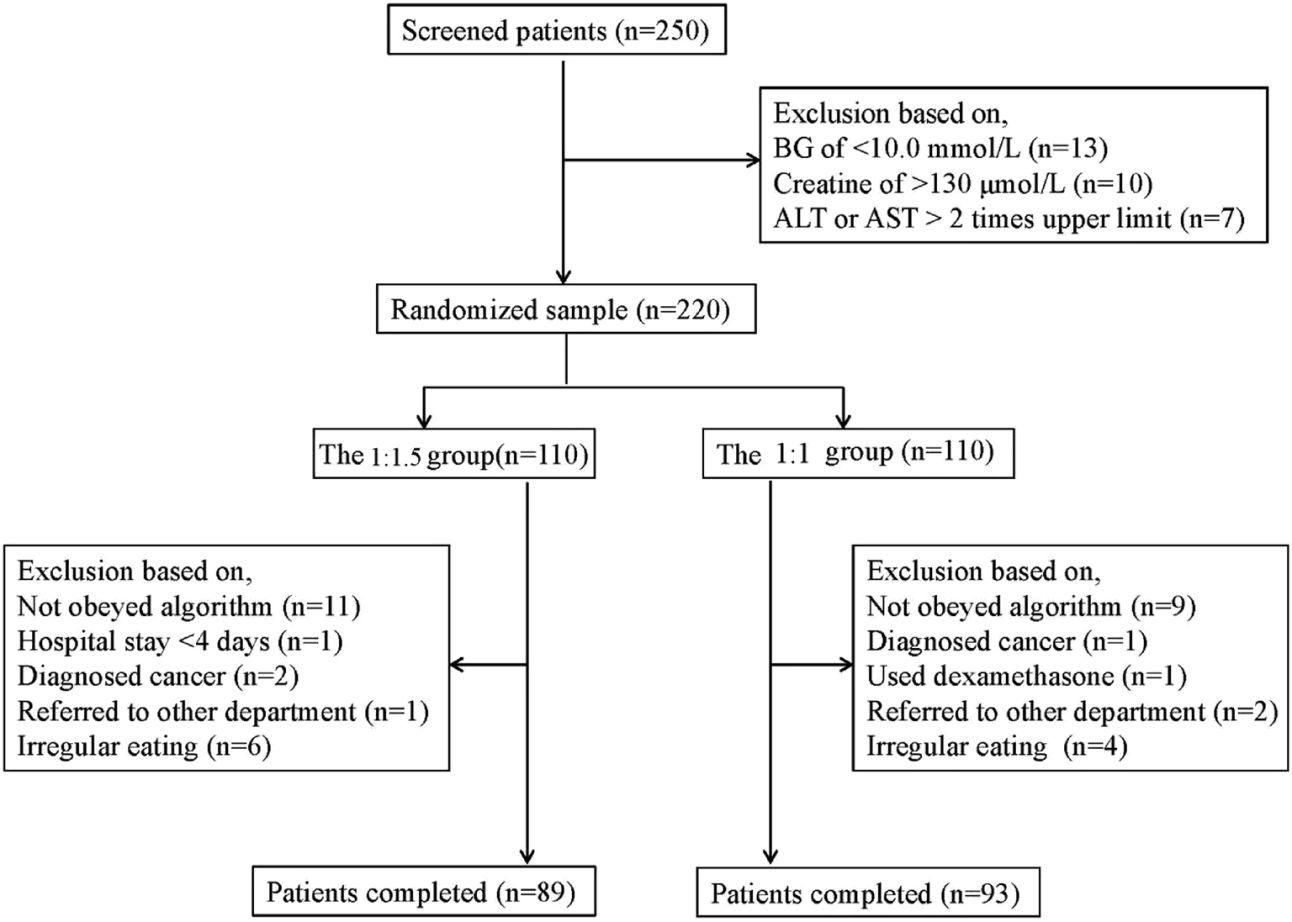
Patients’ selection.  ALT, alanine aminotransferase; AST, aspartate aminotransferase; BG, blood glucose; OHA, oral hypoglycemic agent

Figure 1 shows the patients’ selection. Between October 2021 and June 2022 the number of patients screened was 250. Among them, 220 were selected to randomly assign in equal numbers to the 1:1.5 and 1:1 of glargine to aspart groups. During hospitalization, 21 patients from the 1:1.5 group and 17 from the 1:1 group dropped out of the study. Therefore, 89 patients in the 1:1.5 group and 93 patients in the 1:1 group were analyzed. As shown in Table 2, both groups were well-matched for age, sex, BMI, and initial BG levels. The distribution of previous antidiabetic treatments was also similar in the two groups. The diagnoses on admission included isolated hyperglycemia, diabetic ketoacidosis, and infections.

**Table 2:** Basic characteristics

	1:1.5	1:1
	n=89	n=93
Demographic characteristics		
Sex (M, %)	57 (64.0)	53 (57.0)
Age (years)	57.2 ± 12.8	57.3 ± 10.1
History of diabetes (years)	4.4±5.8	5.8 ±6.9
Weight (kg)	66.8±11.4	65.4±10.8
BMI (kg/m^2^)	25.0±3.3	24.2 ±3.8
Clinical characteristics		
HbA_1c_(%)	11.7±2.1	12.1±1.9
Admission BG (mmol/L)	19.1±5.5	18.2 ±5.3
Diagnosis on admission (%)		
Isolated hyperglycemia	62 (69.7)	64 (68.8)
DK/DKA	15 (16.9)	19 (20.4)
Infection	12 (13.4)	10 (10.8)
Therapy before admission		
None	49 (55.1)	44 (47.3)
OAD	25 (28.1)	29 (31.2)
Insulin	2 (2.2)	2 (2.1)
OAD + insulin	13 (14.6)	18 (19.4)

Values are n (%) or mean ± SD unless otherwise stated. *, median (range). BG, blood glucose. *BMI* body mass index, *DKA* diabetic ketoacidosis. Infection sites include urinary tract, lung, skin, and perianal area. *AGI* alpha-glucosidase inhibitor, *TZD* thiazolidinediones, *DPP4i*, dipeptidyl peptidase 4 inhibitor, *SGLT2i* sodium–glucose co-transporter 2 inhibitor. Sulphonylurea includes gliclazide, glimepiride and glipizide

### Length of time for reaching BG target levels

**Fig. 2:**
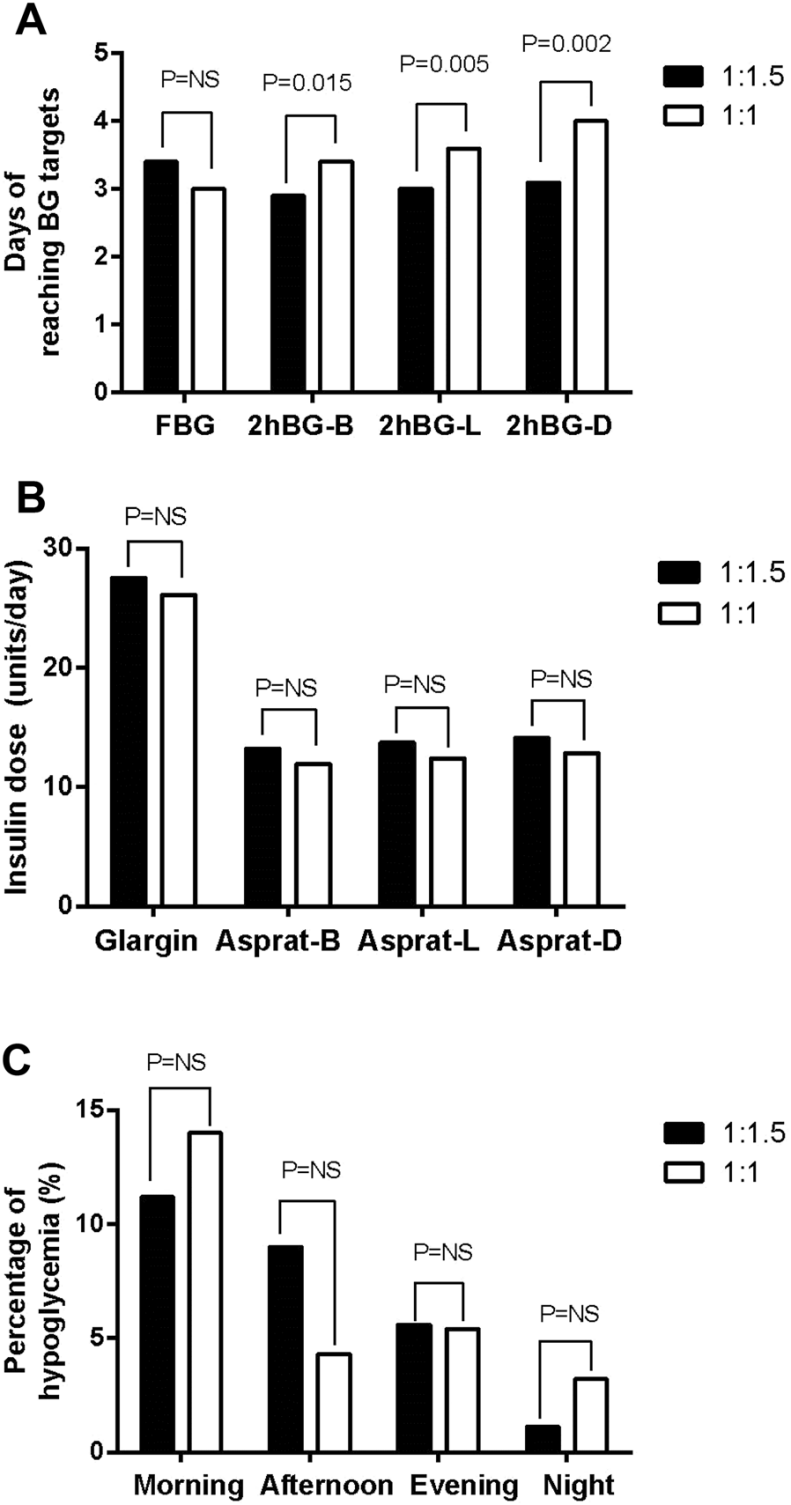
**A** The number of days taken to reach FBG and 2hBG target levels.  B, breakfast; L, lunch; D, dinner. The time taken to reach the FBG target level was not significant between the two groups. The time taken to reach three 2hBG target levels was significant between the two groups. **B **Insulin dosage at the end of treatment.  B, breakfast; L, lunch; D, dinner. The dosage of glargine, pre-breakfast, pre-lunch, and pre-dinner aspart were not significant between the two groups. **C** The percentage of hypoglycemia episode in the 1:1.5 and 1:1 basal-bolus groups

The length of time for reaching the FBG and 2hBG target levels after breakfast, lunch, and dinner in the 1:1.5 group and the 1:1 group were 3.4 ± 1.7 vs. 3.0 ± 1.3 days (p = 0.137), 2.9 ± 1.5 vs. 3.4 ± 1.4 days (p = 0.015), 3.0 ± 1.6 vs. 3.6 ± 1.4 days (p = 0.005), and 3.1 ± 2.1 vs. 4.0 ± 1.5 days (p = 0.002), respectively (Fig. 2A).

Figure 2 A. The number of days taken to reach FBG and 2hBG target levels. B, breakfast; L, lunch; D, dinner. The time taken to reach the FBG target level was not significant between the two groups. The time taken to reach three 2hBG target levels was significant (p = 0.015, 0.005, and 0.002, respectively) between the two groups.

In the 1:1.5 group, 4 patients had two BG levels and 3 patients had one BG level that did not reach the targets. In the 1:1 group, 4 patients had three BG levels and 5 patients had one BG level that did not reach the targets.

### Insulin dosage

The dose of glargine, pre-breakfast aspart, pre-lunch aspart, and pre-dinner aspart in the 1:1.5 group and the 1:1group were 27.5 ± 11.7 vs. 26.1 ± 9.9 units/day (p = 0.339), 13.2 ± 5.6 vs. 11.9 ± 5.0 units/day (p = 0.113), 13.7 ± 6.2 vs. 12.4 ± 4.9 units/day (p = 0.133), and 14.1 ± 6.9 vs. 12.8 ± 5.0 units/day (p = 0.169), respectively (Fig. 2B).

### Hypoglycemia

The percentage of hypoglycemia episodes in the morning (6:30 − 12:00), afternoon (12:00–17:30), evening (17:30 − 23:00), and night (23:00–6:30) in the 1:1.5 group and the 1:1group were 11.2% (10/89. level 1, 8(9.0%); level 2, 2(2.2%)) vs. 14.0% (13/93. level 1, 11(11.8%); level 2, 2(2.2%)), p = 0.578; 9.0% (8/89. level 1, 7(7.9%); level 2, 1(1.1%)) vs. 4.3% (4/93. level 1, 3(3.2%); level 2, 1(1.1%)), p = 0.203; 5.6% (5/89. level 1, 5(5.6%); level 2, 0(0.0%)) vs. 5.4% (5/93, level 1, 4(4.3%); level 2, 1(1.1%)), p = 0.943; and 1.1% (1/89. level 1, 1(1.1%); level 2, 0(0.0%)) vs. 3.2% (3/93. level 1, 3(3.2%); level 2, 0(0.0%)), p = 0.328, respectively (Fig. 2C).

## Discussion

Our previous studies found that weight-based basal and bolus insulin treatment had the same effectiveness and safety as the glucose level-based treatment calculated using an algorithm [16, 19]. Through the present study, using an insulin initiation and titration by weight-based algorithm, a basal-bolus insulin regimen with a fixed dose and ratio of 1:1.5 achieved the BG target levels in a shorter period compared with the 1:1 ratio in hospitalized patients with T2D. The dosages at initiation were 0.2 units/kg for glargine and 0.1 units/kg for aspart to each meal, and those at titration were 0.1 units/kg for glargine and 0.05 units/kg for aspart to each meal daily. Both FBG and 2hBG achieved the target levels in about 3 days. This efficacy was similar to a famous trial that used basal-bolus correction and a glucose-based regimen and achieved mean glucose levels of less than 10.0 mmol/L by day 2 and of less than 8.9 mmol/L by day 4 [13]. The effectiveness of the ratio 1:1.5 could be justified by several explanations. First, patients in this study all had T2D which might have had different pathophysiology compared to the physiological insulin secretion. Also, glargine and aspart are all exogenous insulins. Second, although aspart is considered short-acting insulin, it is sufficient for more than 5 h [20], and the tapering level contributes to the basal insulin. Third, although hospitalized patients in this study had a fixed calorie and fixed proportion of carbohydrates, the Chinese might have preferred more carbohydrates to others [21]. A study conducted with the Latin American non-intensive care unit patients with T2D who used basal-bolus showed a ratio of about 2:3 (glargine 22 units/day and glulisine 31 units/day) to reach a target blood glucose level [18].

In literature and practice, most physicians initiate basal-bolus insulin with a ratio of 1:1 [13–16]. Only in the Johns Hopkins hospital, insulin is initiated in a basal-bolus ratio of 1:1.5 in hospitalized patients [22]. However, the John Hopkins hospital titrates basal bolus based on glucose level which is different from ours which was based on body weight.

Although AACE and ADA recommend basal-bolus insulin instead of slide-scale insulin for glycemic control in hospitalized patients [4], the slide-scale dosage remains the most popular regimen in the majority of hospitals because of its convenience, simplicity, and rapid response [4]. In contrast, the basal-bolus approach requires subcutaneous administration of basal insulin given once daily in combination with prandial and corrective dosages of rapid-acting insulin given before meals. The adoption of the three scales of insulin administration and seven scales of glucose level detection with a correction dosage is very challenging which limits the use by physicians [23]. To overcome inertia, many electronic instruments are designed to assist hospital-based insulin management by clinicians [24–28]. Compared to glucose-based basal-bolus correction regimens and electronic instruments assisting hospital-based insulin management [24–28], our algorithm was cost-effective, simple, and convenient to use.

The present study used glargine other than detemir or degludec. Though detemir was found in an observational study have similar inpatient glycemic control compared with glargine, it was associated with higher daily dose and number of injections [29]. Degludec was non-inferiority to glargine in hospitalized patients with T2D [30]. However, in some studies, due to its prolonged effect of more than 24 h, the quantity was adjusted every 2 days instead of once daily which may prolong the length of stay [31]. Short-acting insulin, though there is no head-to-head trial, glulisine, aspart and lispro had been widely studied as effective and safe using basal-bolus algorism in inpatients [13, 16, 19, 32, 33]. Faster aspart and faster lispro need evidence for inpatients.

Hypoglycemia is one of the main concerns during antidiabetic treatment, especially insulin treatment. Our hypoglycemia episode rate of 26.9% in the 1:1.5 group was similar to other basal-bolus randomized control trials which had 16.0–35.0% [13, 18, 33–36]. A clinical trial conducted in China using basal-bolus insulin also showed a similar hypoglycemia rate of 28.0% [36]. No severe hypoglycemia was found in this trial. In this regard, 1:1.5 basal-bolus insulin initiation and titration using a weight-based algorithm were safe for hospitalized patients with T2D.

Nevertheless, the present open-label study also had several limitations. First, all patients included in the study were on regular diets and had normal renal and liver functions. Therefore, whether the algorithm is applicable to patients with parenteral nutrition, and renal and liver dysfunction remains to be elucidated. Second, the subjects included in this study came from the same ethnic origin and the mean BMI values of these patients were relatively low (< 25 kg/m^2^). Third, most patients in this study had higher hyperglycemia on admission (mean HbA1c > 10%). Therefore, clinical trials with other populations and patients with relatively lower glycemic levels are required to determine the efficacy and safety in those groups using the new algorithm.

## Conclusion

We demonstrated that fixed dose-ratio basal-bolus insulin at 1:1.5 calculated using a weight-based initiation and titration algorithm was simple, as effective, and safe as ratio at 1:1 in managing T2D in hospitalized patients.

## Data Availability

The datasets generated during and/or analyzed during the current study are not publicly available but are available from the corresponding author on reasonable request.
